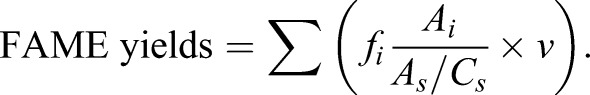# Identification and characterization of a symbiotic alga from soil bryophyte for lipid profiles

**DOI:** 10.1242/bio.019992

**Published:** 2016-08-19

**Authors:** Jia Feng, Yuning Guo, Xiujuan Zhang, Guihua Wang, Junping Lv, Qi Liu, Shulian Xie

**Affiliations:** School of Life Science, Shanxi University, Taiyuan 030006, China

**Keywords:** Symbiotic, Bryophyte, *Chlorococcum sphacosum*, Phylogenetic analysis, Lipid accumulation

## Abstract

A symbiotic alga was successfully isolated from the soil moss *Entodon obtusatus* found in the Guandi Mountains, Shanxi Province, China, and cultivated under axenic conditions. Morphological observations showed that the symbiotic alga was similar to *Chlorococcum*. Based on phylogenetic analysis of 18S rRNA and *rbc*L genes and internal transcribed spacer (ITS) regions, *Chlorococcum* sp. GD was identified as *Chlorococcum sphacosum*. The three data sets were congruent for those aspects of the topologies that were relatively robust, and differed for those parts of the topologies that were not. This strain was cultured in BG11 medium to test its growth and biodiesel properties. It produced a lipid content of nearly 40%, and achieved biomass concentration of 410 mg l^−1^ and lipid productivity of 6.76 mg l^−1^ day^−1^, with favorable C16:0 (23.10%) and C18:1 (21.62%) fatty acid content. This alga appears to have potential for use in biodiesel production.

## Abstract (precis)

**Summary:** A symbiotic alga *Chlorococcum* sp. GD was successfully isolated from the soil moss and successfully cultivated under axenic conditions. It has nearly 40% lipid content, and thus appears to have potential for use in biodiesel production.

## INTRODUCTION

Energy crises and global warming demand the development of biofuels as renewable alternatives to, and replacements for, fossil fuels (Hu et al., 2008; Mata et al., 2010; Hoekman et al., 2012). Microalgae are considered to have great potential as a substitute for fossil fuels because of their high photosynthetic efficiency, biomass production and growth rates compared with other energy crops (Hoekman et al., 2012; Miao and Wu, 2006; Xin et al., 2010; Sivakumar et al., 2012). Consequently, microalgae-based biodiesel has attracted more and more attention; therefore, we need to identify many more microalgal species which could be exploited as bio-resources (Grobbelaar, 2000).

Selection of highly productive, oil-rich algal strains is fundamentally important to enrich the algal collection available for biodiesel production (Griffiths et al., 2012). Several high-oil algal strains have been reported, such as *Nannochloropsis* sp., *Botryococcus braunii*, and *Chlorella* sp. (Li et al., 2011a,b; Lv et al., 2010), which can have a total lipid content of 30–60% of their dry weight (Chisti, 2007).

The specific objectives of this study were to isolate and identify robust microalgal strains from freshwater habitats, to characterize the selected strains for their biomass and lipid production, and to determine the most promising strains with high oil-production and suitable fatty acid composition profiles for biofuel production. A microalgal strain with high lipid accumulation was obtained fortuitously during research on the symbiotic relationships between bryophytes and algae.

## RESULTS AND DISCUSSION

### Morphological features of the symbiotic alga

**Fig. 1.**
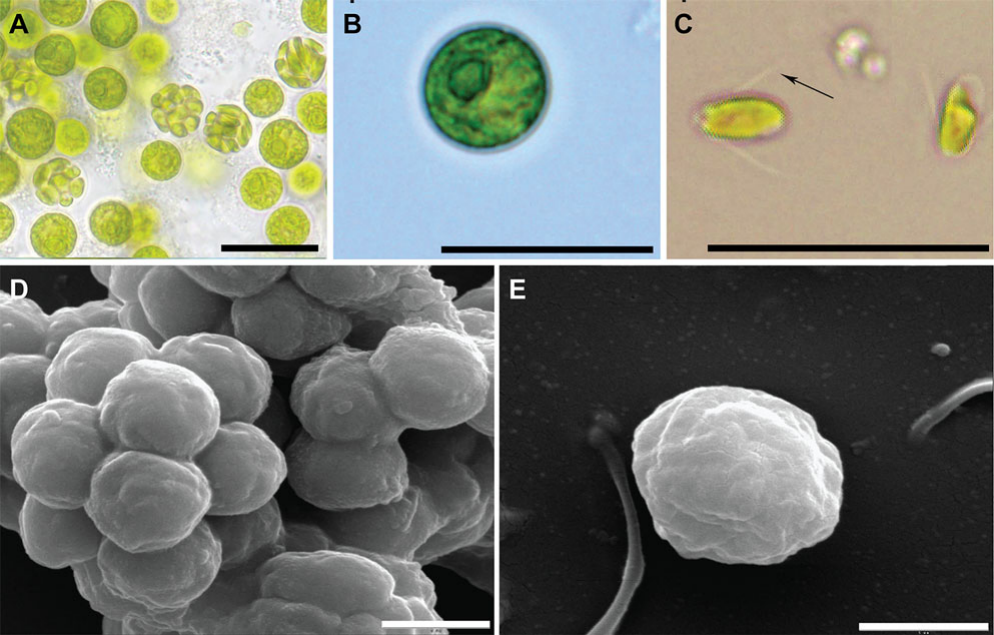
**Morphology of the symbiotic algae *Chlorococcum* sp. GD.** (A) Light microscopic images of cell morphology of the symbiotic algae *Chlorococcum* sp. GD, mature strains divided into zoospores (culture 9 days). (B) Cell spherical or ovoid (culture 5 days). (C) Zoospores with two equal flagella; arrow indicates the flagella of the cell. (D) Scanning electron microscope images of cell morphology of the symbiotic algae *Chlorococcum* sp. GD. (E) Single cell of symbiotic algae *Chlorococcum* sp. GD. Scale bars: 200 μm for A, B and C, 5 μm for D and E.

The bryophyte specimens collected were identified as *Entodon obtusatus* (Entodontaceae) based on macro- and micro-morphological features (Fig. S1) (Zhu et al., 2002). Voucher specimens (No. SAS2013018) were deposited in the herbarium of Shanxi University (SXU). Vegetative cells of the symbiotic algae appear ellipsoidal to spherical and vary in size in the light microscope image. Young cell walls are thin and smooth and become thicker with growth. Chloroplasts are parietal, with or without a peripheral opening, and contain one pyrenoid. Motile cells have two equal flagella and remain ellipsoidal for a time after motility ceases. Eight to 32 aplanospores or zoospores are present, forming an aplanosporangium or zoosporangium, respectively. Zoospores are spindle-shaped to ovoid, with two flagella (Fig. 1A-C).

Under the scanning electron microscope, cells appear spherical to spindle-shaped. The cell wall has an irregular rib net, which differs from the smooth surface observed under the light microscope (Fig. 1D,E).

**Fig. 2.**
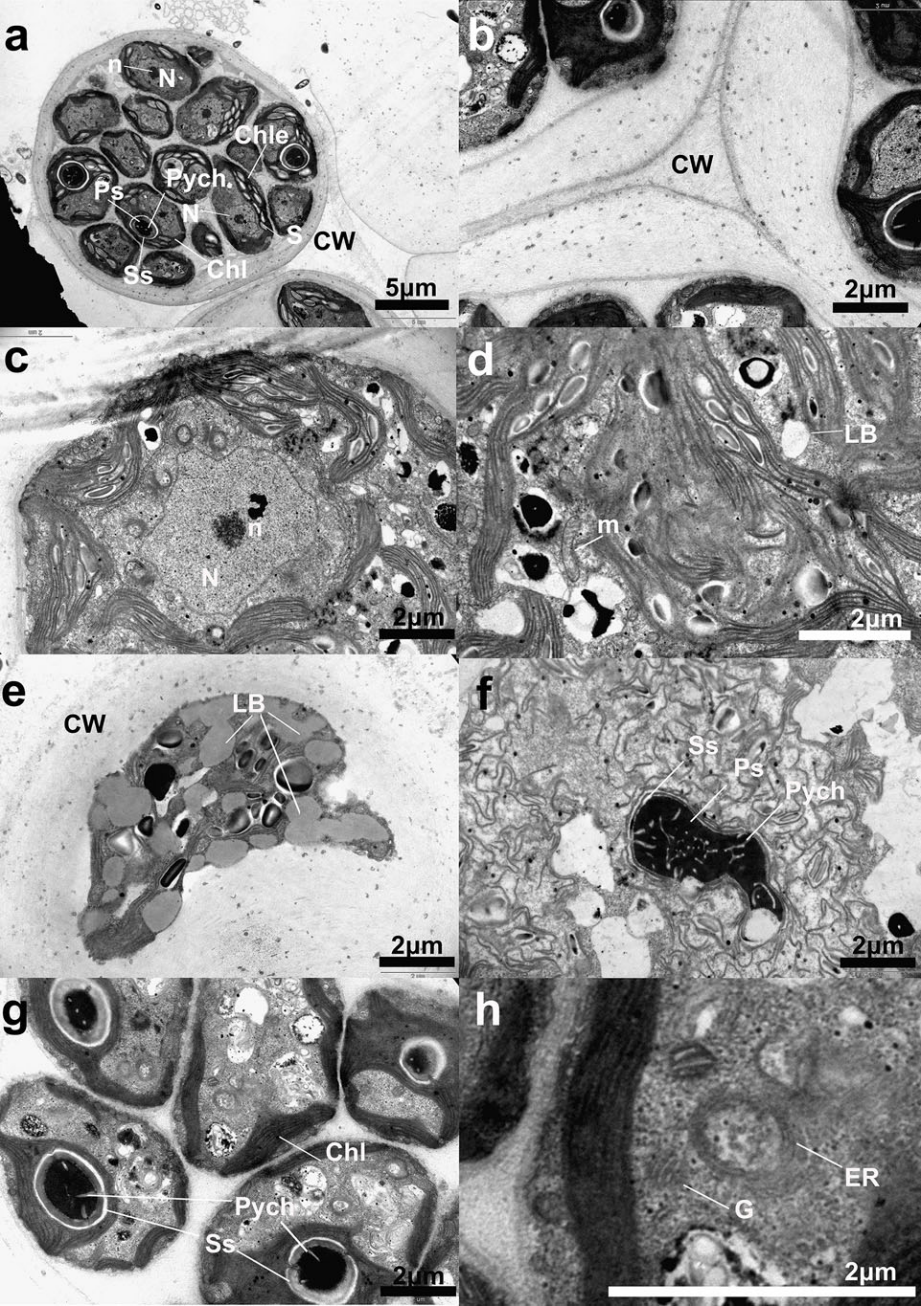
**Transmission electron microscope images of cell morphology of the symbiotic algae *Chlorococcum* sp. GD.** (A) Vegetative cell ×6000. (B) Cell wall ×15,000. (C) Nucleus ×15,000. (D) Mitochondria ×15,000. (E) Lipid droplets ×15,000. (F) Pyrenoid ×15,000. (G) Chloroplast, pyrenoid ×15,000. (H) Golgi body, endoplasmic reticulum, ×15,000. CW, cell wall; Chle, chloroplast envelope; Chl, chloroplast lamellae; ER, endoplasmic reticulum; G, Golgi body; LB, lipid droplet; m, mitochondria; N, nucleus; Ps, pyrenoid matrix; Pych, intrapyrenoidal channels lined with double membranes originating from the chloroplast; S, starch grains; Ss, starch sheath.

Two layers of cell wall and the trilaminar membrane were observed under the transmission electron microscope, as well as the nucleus, mitochondria, Golgi bodies, endoplasmic reticulum, and chloroplasts. The chloroplast forms a hollow ball, occupying a large fraction of the total cell volume. Thylakoids form long plates composed of fascicles, distributed with electron-dense small particles and irregularly shaped starch granules, which differs from the grana stacking seen in the chloroplasts of higher plants. A large pyrenoid has a continuous starch sheath of uniform thickness in the thick basal part of the chloroplast. The nucleus is posterior to the pyrenoid. Mitochondria have a structure typical of green algae, composed of a double membrane with flat cristae distributed across the inner membrane. The lipid droplet is clearly visible with electron-transparent contents (Fig. 2).

Based on morphological characters, the symbiotic algae resemble *C. sphacosum* in plant mass appearance but differ in rough surface. *C. sphacosum* was first isolated from a *Sphagnum* bog near Falmouth, Barnstable County, Massachusetts by Archibald and Bold (1970), and was named in reference to its habitat. These symbiotic algae were thus preliminarily identified as *Chlorococcum* sp. GD (Guandi Mountains), and belong to the Chlorophyceae (Starr, 1955; Péterfi et al., 1988).

### Phylogenetic analyses

To further identify the taxonomic position of *Chlorococcum* sp. GD, molecular phylogenetic analysis was carried out. The species in each phylogenetic analysis were used depending on the data availability in the gene database NCBI GenBank (http://www.ncbi.nlm.nih.gov/genbank/).

**Fig. 3.**
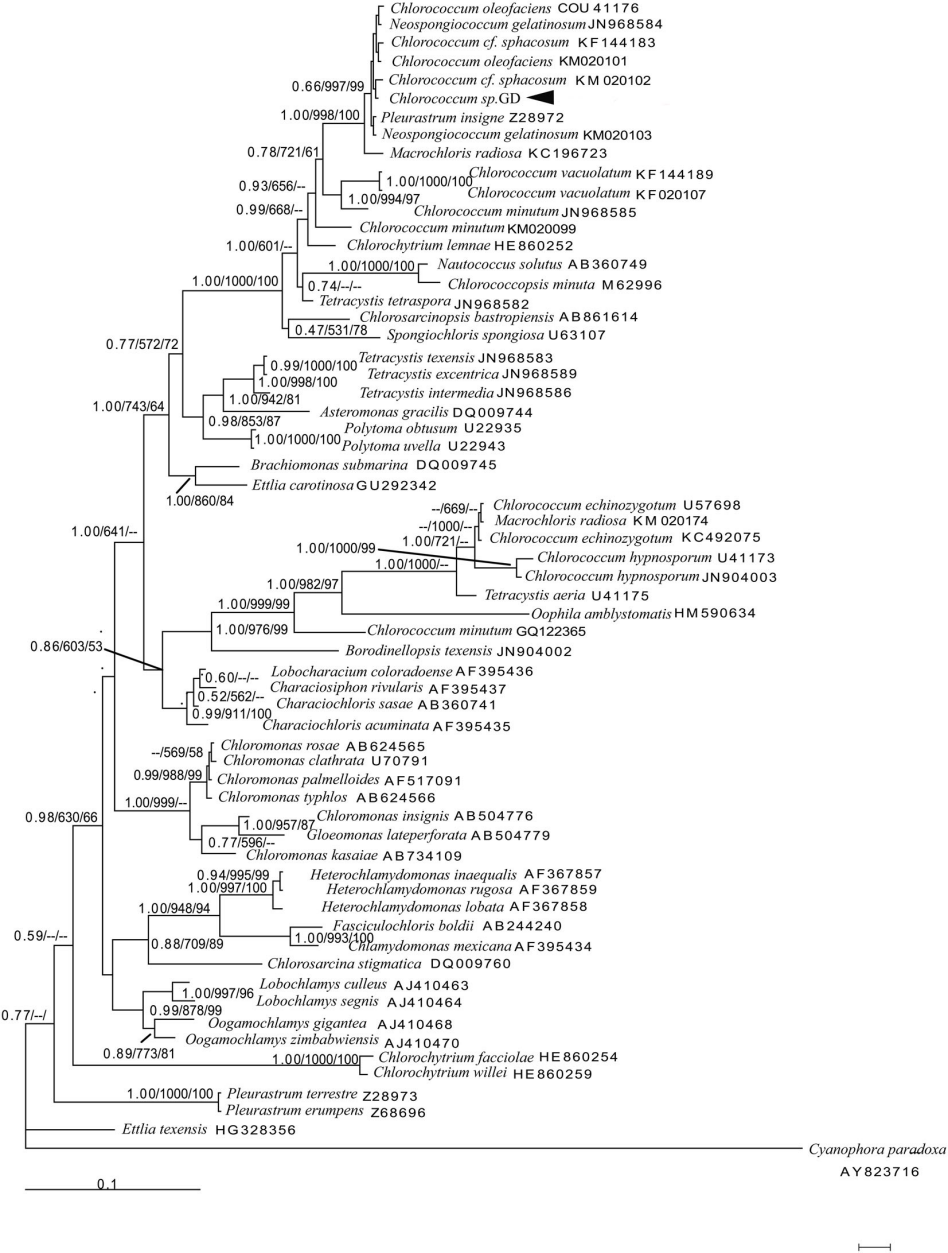
**Hypothesized phylogenetic relationships based on BI analysis of the 18S rRNA gene of *Chlorococcum* sp. GD and other species.** Support values for individual branches are given as Bayesian posterior probability/ML bootstrap/NJ bootstrap. Values <50% are not displayed.

**Fig. 4.**
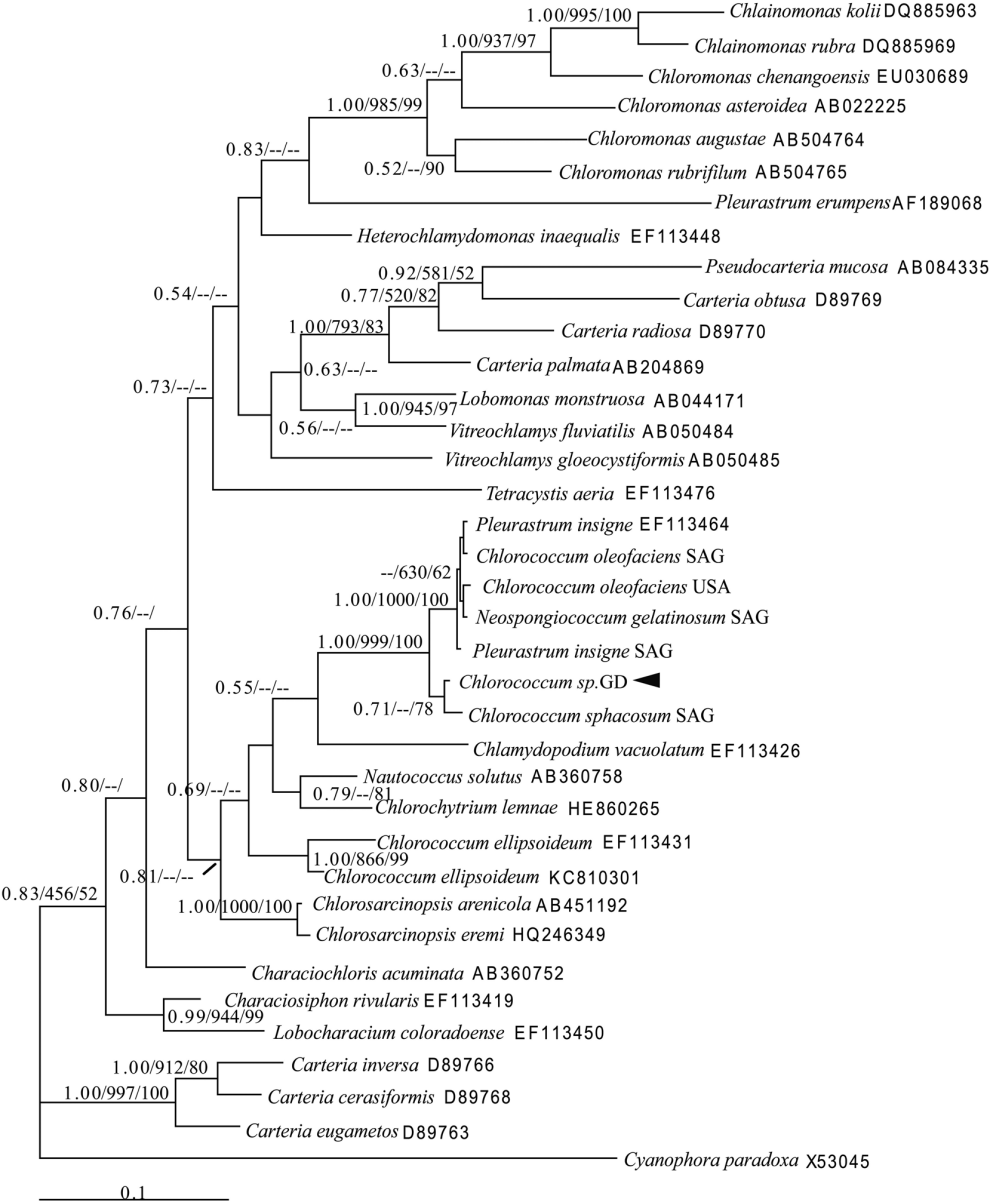
**Hypothesized phylogenetic relationships based on BI analysis of the *rbcL* gene of *Chlorococcum* sp. GD and other species.** Support values for individual branches are given as Bayesian posterior probability/ML bootstrap/NJ bootstrap. Values <50% are not displayed.

**Fig. 5.**
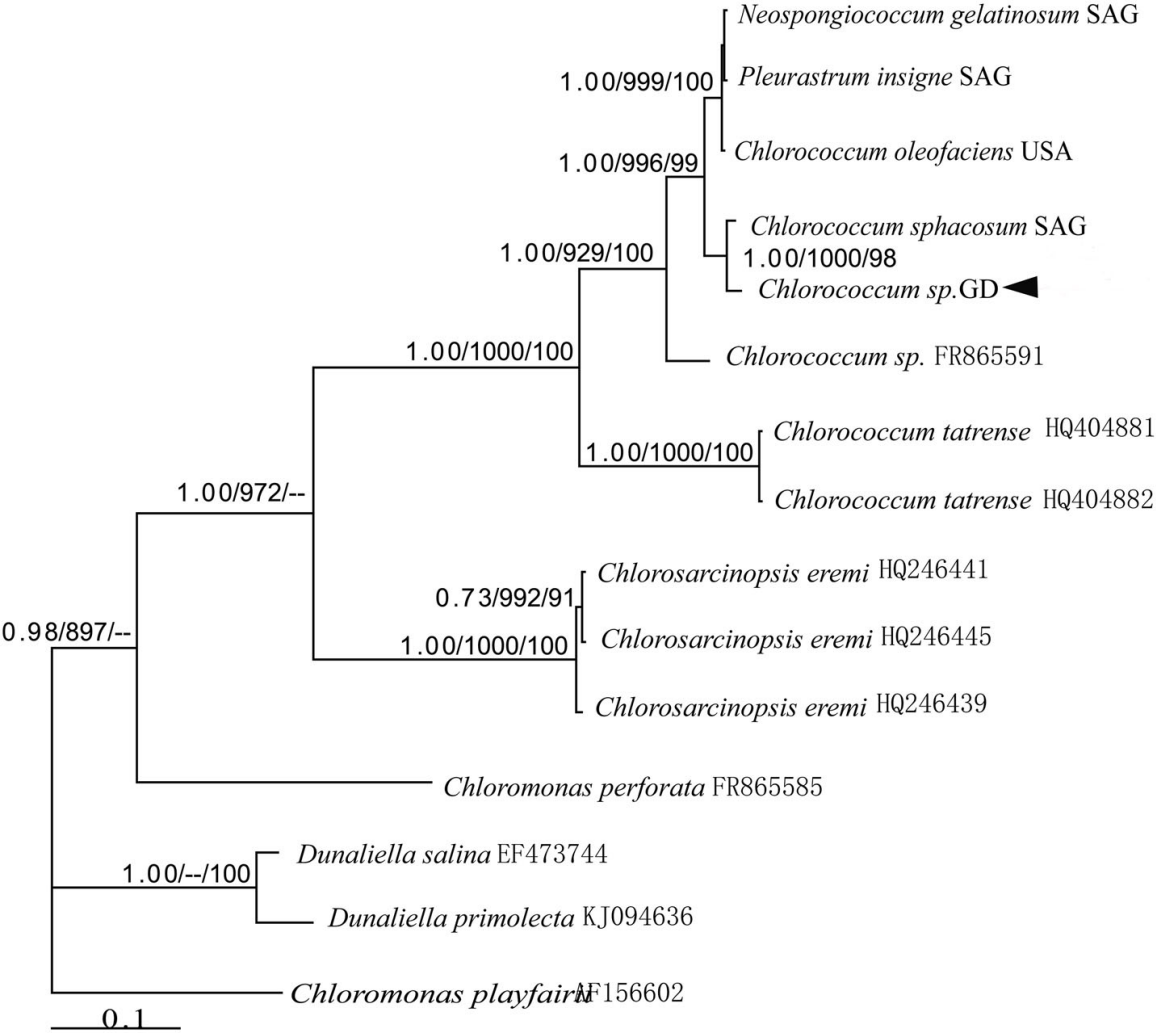
**Hypothesized phylogenetic relationships based on BI analysis of the ITS gene of *Chlorococcum* sp. GD and other species.** Support values for individual branches are given as Bayesian posterior probability/ML bootstrap/NJ bootstrap. Values <50% are not displayed.

PCR amplification of 18S rRNA, *rbcL*, and ITS regions of *Chlorococcum* sp. GD produced 1363 bp, 649 bp, and 633 bp amplicons, respectively. The sequences were analyzed using Modeltest 3.7 to determine the best-fitting models of sequence evolution, and these are listed in Table S1. Maximum likelihood (ML), neighbor joining (NJ), and Bayesian inference (BI) trees based on each DNA region were constructed from individually aligned datasets comprising sequences of *Chlorococcum* sp. GD and other strains. For the three alignments, topologies recovered based on the BI algorithm are shown in Figs 3–5, respectively, with ML and NJ bootstrap support values indicated.

In the phylogenetic tree generated by BI analysis of 18S rRNA sequences (Fig. 3), *Cyanophora paradoxa* is the outgroup. *Chlorococcum* sp. GD is grouped together with *C. cf. sphacosum* (KM020102), *C. oleofaciens* (KM020101), *C.* cf. *sphacosum* (KF144183), *Neospongiococcum gelatinosum* (JN968584), *C. oleofaciens* (COU41176), *Pleurastrum insigne* (Z28972), and *N. gelatinosum* (KM020103) in a well-supported clade (ML bootstrap value/NJ bootstrap value/BI posterior probability of 0.66/99.7%/99%).

The tree recovered by BI analysis of *rbcL* sequences is shown in Fig. 4. *Chlorococcum* sp. GD is sister to strain *C. sphacosum* SAG (ML bootstrap value/NJ bootstrap value/BI posterior probability of 71%/0/78%). These two strains constitute a clade (BI/ML/NJ support=1.00/99%/1.00) together with *P. insigne* SAG, *N. gelatinosum* SAG, *C. oleofaciens* USA, *C. oleofaciens* SAG, and *P. insigne* (EF113464).

The unrooted phylogenetic tree recovered by BI analysis of ITS sequences is shown in Fig. 5. *Chlorococcum* sp. GD is sister to strain *C. sphacosum* SAG (ML bootstrap value/NJ bootstrap value/BI posterior probability of 1.00/1.00/98%). These two strains constitute a clade (BI/ML/NJ support=1.00/99.6%/99%) together with *P. insigne* SAG, *N. gelatinosum* SAG, and *C. oleofaciens* SAG.

Taxonomic problems in the Chlorophyceae have been successfully addressed by many previous researchers using DNA sequence data (Fawley et al., 2011). Pre-genomic tools such as ribosomal RNA (rRNA) genes, the sequences of which are highly conserved, can be used with or without full genomic information for investigating microbial communities (Rittmann et al., 2008). The sequence information for the 18S rRNA and *rbcL* genes suggest that the genus *Chlorococcum* is not monophyletic but polyphyletic, with some strains belonging to the *Macrochloris* clade and to the *Tetracystis* clade. *Chlorococcum* sp. GD is related to *C.* cf. *sphacosum* and *C. sphacosum*. Compensatory base changes (CBCs) in the internal transcribed spacer region (ITS) of the nuclear rRNA cistron have been suggested for use as molecular classifiers, to indicate whether two organisms belong to different species (Müller et al., 2007). The phylogenetic tree based on the ITS region confirmed the close affinity of *C. sphacosum*. The three data sets were congruent for those aspects of the topologies that were relatively robust, and differed for those parts of the topologies that were not (Kawasaki et al., 2015).

### Growth and lipid accumulation properties of *Chlorococcum* sp. GD

**Fig. 6.**
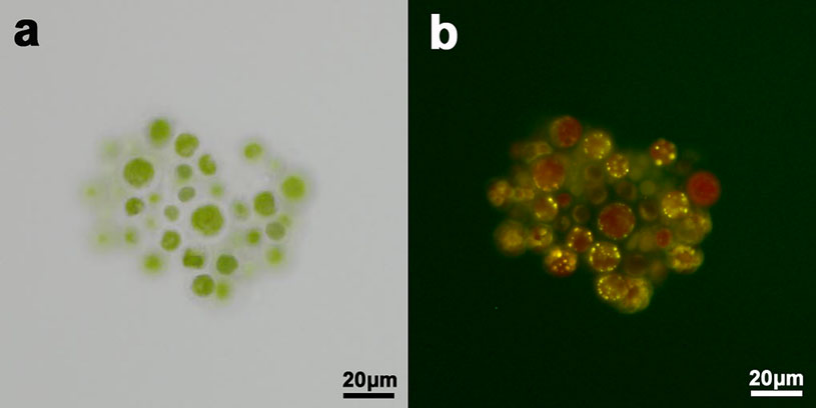
**Microscopic photos of *Chlorococcum* sp. GD before and after stained by Nile red.** (A) Photomicroscope observation of *Chlorococcum* sp. GD before stained by Nile red. (B) Intense fluorescence was observed in *Chlorococcum* sp. GD after stained by Nile red.

To determine the promising algal strain for lipid production, it displayed obvious lipid accumulation in culture cell stained with Nile red (Fig. 6) by fluorescence microscopy.

**Fig. 7.**
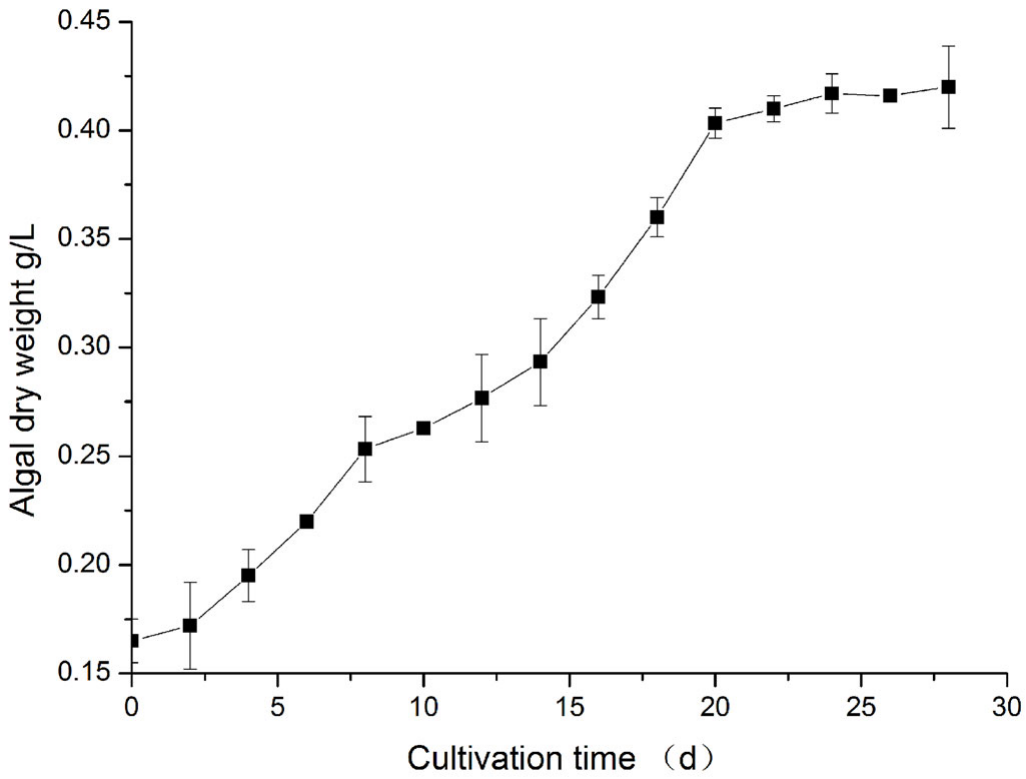
**Growth curves** **of *Chlorococcum* sp. GC cultured in BG-11.** The Growth curve was measured by algal dry weight per liter culture every two days. Each data point has three biological repeats. 0-22 days were exponential phase, while after 22 days the algal growth entered in stable phase.

To further evaluate biomass and lipid production, *Chlorococcum* sp. GD was cultured in BG11 medium. The growth curve and dry weight of *Chlorococcum* sp. GD are shown in Fig. 7. On day 22 of cultivation, the microalga grew into a stable phase. It produced a lipid content of nearly 40%, which is higher than the average value of 20% reported for other algal species (Rodolfi et al., 2009; Li et al., 2011a,b) under the same cultivation conditions. This strain cultivated for 22 days and achieved a biomass concentration (DM) of 410 mg l^−1^, the specific growth rate (k) of 0.0413 day^−1^, and a lipid productivity (PL) of 6.76 mg l^−1^ day^−1^, compared with other green algal strains with lipid productivities of 0.8-9 mg l^−1^ day^−1^ (Song et al., 2013).

### Fatty acid properties of *Chlorococcum* sp. GD

**Fig. 8.**
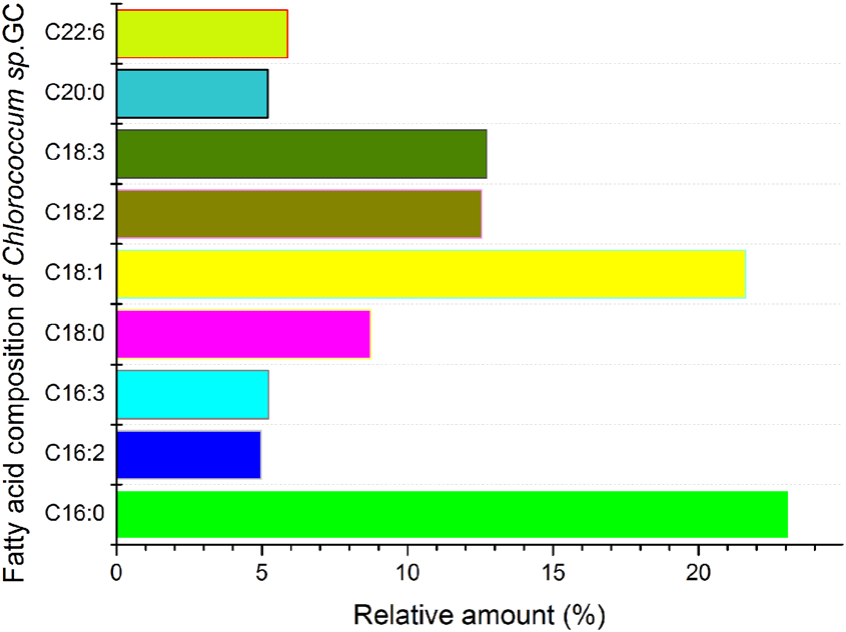
**Fatty acid composition of *Chlorococcum* sp. GC.** All fatty acid components were analyzed by GC-MS, the relative amount indicates the abundance of certain component in total fatty acid.

In the present study, we have characterized a symbiotic alga and screened it for a fatty acid composition conforming to ATSM D6751 (US) and UNE-EN 14214 (EU) international biodiesel standards. It is important to characterize the fatty acid profile when proposing the utility or potential of microalgal lipids as biodiesel feedstock oil. We determined the total lipid content and fatty acid compositions of the isolated microalgal species *Chlorococcum* sp. GD. The fatty acid methyl ester (FAME) compositional profiles are shown in Fig. S2. The gas chromatography–mass spectrometry (GC–MS) analysis shows that the main fatty acids are C16–C18, which are commonly found in feedstock suitable for biodiesel production (Knothe, 2009). The major fatty acids found in the microalga were palmitic acid (C16:0), oleic acid (18:1), linolenic acid (18:3) and linoleic acid (18:2) (Fig. 8). Freshwater microalgae synthesize C16:0, C18:1, C18:2, and C18:3 fatty acids with species-specific relative concentrations of other fatty acids (Kaur et al., 2012). Oleic acid (18:1) is considered the optimum fatty acid for biodiesel, as it gives the finest compromise between oxidative stability and cold flow properties (Hoekman et al., 2012; Knothe, 2008).

### Conclusions

The symbiotic alga was identified by its morphological features as *Chlorococcum* sp. GD associated with *E. obtusatus* (Entodontaceae). Phylogenetic trees based on 18S rRNA, *rbc*L and ITS regions confirmed its close affinity to *C. sphacosum*. The isolated *Chlorococcum* sp. GD produced a lipid content of nearly 40% and achieved high lipid productivity. The fatty acid profile is rich in C16 and C18, which are commonly found in feedstock suitable for biodiesel production. These results indicate that the alga has great potential for use in biodiesel production.

## MATERIALS AND METHODS

### Sample collection and processing

Bryophyte specimens were collected from the Bashuigou landscape of the Guandi Mountains, Shanxi Province, northern China, in July 2013 (37°49′N, 111°27′E, altitude 1861 m).

Fresh stems and leaves of bryophytes were washed under running water for 15 min and then three times under distilled water. The surfaces of selected bryophyte thalli were cleaned repeatedly with a soft brush on a clean bench, washed with Tween20 (Dengfeng Chemical Co., Tianjin) and then washed five times with ultrapure water. The fifth wash of ultrapure water was collected as a control to test whether the thallus surfaces were clean (Zhang and Shi, 2010; Guo et al., 2012). The cleaned thalli were homogenized in a sterile mortar. The homogenates were transferred to BG-11 liquid medium in 100 ml conical flasks, then cultured in a light incubator (BSG-300, Boxun, China) at 20°C under a 12 h:12 h light:dark regime. The light intensity was 25 µmol m^−2^ s^−1^. The ultrapure water from the final wash was transferred to BG-11 liquid medium and cultured in the same conditions as the algae. After 20 days of culture, the cultured ultrapure water was examined under a light microscope every 5 days (BX-51, Olympus, Tokyo, Japan).

After 3–4 weeks of culture, green algae were observed to grow on residual bryophyte fragments. Six strains were isolated following standard methods (Ban et al., 2013) and cultured on BG-11 liquid medium in 24-well cell culture plates at 25°C under a 12 h:12 h light:dark regime.

To further identify the taxonomic position of the symbiotic alga, related strains (determined on the basis of morphological characters) were obtained from either the Sammlung von Algenkulturen (Collection of Algal Cultures) at the University of Göttingen (SAG, http://www.uni-goettingen.de/en/184982.html) or the Culture Collection of Algae at the University of Texas at Austin (UTEX, http://web.biosci.utexas.edu/utex/). *Chlorococcum oleofaciens* (UTEX 105), *Pleurastrum insigne* (SAG 30.93), *Neospongiococcum gelatinosum* (SAG 64.80), *C. oleofaciens* (SAG 213-11) and *C. Sphacosum* (SAG 66.80) (Kawaida et al., 2013) were also cultured on BG-11 liquid medium in 24-well cell culture plates at 25°C under a 12 h:12 h light:dark regime.

### Morphological observations

The morphological features of the bryophytes and associated algae were observed and photographed under a light microscope (BX-51, Olympus, Japan). Photographs of the main characteristics were taken with a digital camera (CAMEDIA C5060WZ, Olympus, Japan) and a charge-coupled device (DP72, Olympus, Japan).

The cell morphology of the isolated and cultured strain was observed using a scanning electron microscope (S-3500N, Hitachi, Japan) and a transmission electron microscope (JEOL-1200EX, Hitachi, Japan).

The lipid accumulation of the isolated and cultured strain was observed using fluorescence microscopy (BX41, Olympus, Japan).

### DNA extraction, PCR amplification and sequencing

Total DNA was extracted from the cultured filaments using a plant DNA extraction kit (Sangon Biotech, Shanghai, China). For polymerase chain reaction (PCR) amplification of the 18S rRNA region, we used primers MA1 (5′-CGGGATCCGTAGTCATATGCTTGTCTC-3′) and MA2 (5′-CGGAATTCCTTCTGCAGGTTCACC-3′) (Olmos et al., 2000). The chloroplast *rbcL* gene region was amplified using primers 475–497 (5′-CGTGACAAACTAAACAAATATGG-3′) and 1181–1160 (5′-AAGATTTCAACTAAAGCTGGCA-3′) (Nozaki et al., 1997). The nuclear rDNA ITS region (ITS-1+5.8S rDNA+ITS-2) was amplified using universal primers AB28 (5′-GGGATCCATATGCTTAAGTTCAGCGGGT-3′) and TW81 (5′-GGGATCCGTTTCCGTAGGTGAACCTGC-3′). PCR amplifications were performed in 20 µl reaction volumes containing 12.8 µl double-distilled water, 2.5 µl 10× Taq polymerase reaction buffer (Takara, Dalian, China), 0.2 µl Taq DNA polymerase, 1 µl of each 10 µM primer, 2 µl dNTP mix (2.5 mM each; Takara), and 1 µl of undiluted genomic DNA. Amplifications were performed in a MyCycler thermal cycler (Bio-Rad, Hercules, CA, USA). PCR amplifications of 18S rRNA, *rbcL* and ITS (ITS-1+5.8S rDNA+ITS-2) regions were carried out as follows: initial denaturation at 95°C for 5 min, followed by 35 cycles of 94°C for 45 s, 55°C for 45 s, and 72°C for 1 min, and a final extension step of 72°C for 10 min. PCR products were purified using a Gel Extraction Mini Kit (Watson) according to the manufacturer's instructions. After purification, PCR products were sequenced by Sangon Biotech Company (Shanghai, China). Sequences generated from the research were deposited in GenBank (identified by asterisks and under accession numbers listed in Table S2).

### Sequence alignment and phylogenetic analyses

Sequences were aligned using ClustalX 2.0 (Thompson et al., 1997) and then manually adjusted. Phylogenetic trees were constructed from the aligned gene sequences using neighbor-joining (NJ), maximum likelihood (ML), and Bayesian (BI) methods. NJ, ML, and BI analyses were performed using MEGA 5.0 (Tamura et al., 2011), PhyML 3.0 (Guindon and Gascuel, 2003), and MrBayes 3.1.2 (Ronquist and Huelsenbeck, 2003), respectively. The program ModelTest 3.7 (Posada, 2008) was used to determine the best-fitting models of sequence evolution for ML and BI methods. For the analysis of 18S rRNA and *rbcL*, *Cyanophora paradoxa* was selected as the outgroup.

### Lipid determination

Microalgal growth was measured every 24 h based on enumeration as described by Ban et al. (2013). Specific growth rate per day was calculated according to the following equation:
(1)
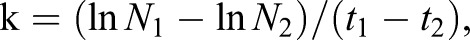
where k (day^−1^) is the specific growth rate in exponential growth phase, and *N_1_* and *N_2_* are the biomass concentrations at day *t_1_* and *t_2_*. Biomass productivity was obtained according to the following calculation (Song et al., 2013):
(2)
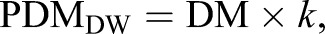
where PDM_DW_ is the biomass productivity (mg l^−1^ day^−1^) in the exponential growth phase and DM (mg l^−1^) is the biomass concentration at the end of the exponential phase. The total lipid contents were extracted with a chloroform/methanol (2:1, v/v) mixture and were quantified gravimetrically (Song et al., 2013). The lipid productivity (PL) was calculated according to following equation:
(3)
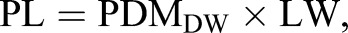
where PL is the lipid productivity (mg l^−1^ day^−1^) in the exponential growth phase, and LW represents lipid content based on dry weight.

Fatty acid content and composition were determined in two steps: preparation of fatty acid methyl esters (FAME) and gas chromatography–mass spectrometry (GC–MS) analysis. FAME was prepared by acid-catalyzed esterification (Song et al., 2014). The algal samples were dissolved in chloroform, and then reacted directly with 1.0 ml of a mixture of methanol and sulfuric acid (1 mol/l) in Agilent bottles. Transesterification was carried out in a 100°C water bath for 1 h and then 200 μl ultrapure water was added. Upon completion of the reaction, hexane (200 μl) was added to the solution, which was homogenized (Ultrasonic Homogenizer SCIENTZ-IID, China) for 10 min, and then separated into two layers. Heptadecanoic acid methyl ester (50 µl, 2 mg ml^−1^) was added to the upper phase (200 µl) for methyl ester analysis on GC–MS (7890-N5973, Agilent, USA). Solution (1 μl) was injected in splitless mode and the injector temperature raised from 50°C to 150°C and held for 2 min, then raised to 200°C at a rate of 10°C min^−1^ and held for 6 min. The oven temperature was further raised to 230°C at a rate of 10°C min^−1^, held for 30 min, then raised to 240°C at a rate of 10°C min^−1^, and held at 240°C for 10 min. Mass spectra were recorded under electron ionization (70 eV) at a frequency of 5 scans for 1 s. The ion source temperature was 240°C, and a full scan was carried out over the range of 20–450 amu. In order to calculate the FAME yield of samples, a mixture of FAME standards (Sigma Aldrich 47,885, 10 mg ml^−1^) was analyzed under the same GC–MS conditions described above. FAME yields were calculated using Eqn (4), where *f_i_* is a correction factor for section i obtained from analysis of the mixture of FAME standards, *A_i_* is the peak area of section i, *A_s_* is the peak area of the internal standard, *C_s_* is the concentration of the internal standard and *v* is the volume of the organic phase (Song et al., 2014):
(4)